# Editorial: Bicuspid aortic valve: from pathophysiological mechanisms, imaging diagnosis to clinical treatment methods

**DOI:** 10.3389/fcvm.2023.1193544

**Published:** 2023-04-28

**Authors:** Yi Zhang, Tian-Yuan Xiong, Lars Sondergard, Darren Mylotte, Nicolo Piazza, Bernard Prendergast, Mao Chen

**Affiliations:** ^1^ Department of Cardiology, West China Hospital, Sichuan University, Chengdu, China; ^2^ The Heart Center, Rigshospitalet, University of Copenhagen, Copenhagen, Denmark; ^3^ Department of Cardiology, University Hospital Galway, National University of Ireland, Galway, Ireland; ^4^ Division of Cardiology, Department of Medicine, McGill University Health Center, Montreal, QC, Canada; ^5^ Department of Cardiology, St Thomas’ Hospital, London, United Kingdom; ^6^ Cleveland Clinic London, London, United Kingdom

**Keywords:** bicuspid aortic valve, valvular heart disease, aortopathy, aortic dilatation, transcatheter aortic valve replacement

**Editorial on the Research Topic** Bicuspid aortic valve: from pathophysiological mechanisms, imaging diagnosis to clinical treatment methods

This Research Topic, entitled “Bicuspid Aortic Valve: From pathophysiological mechanisms, imaging diagnosis to clinical treatment methods”, is created to set a forum for researches that tackle the difference or uniqueness of the BAV entity, from genetic, cellular and pathophysiological mechanisms of BAV and the subsequent bicuspid aortic stenosis, clinical imaging for bicuspid aortic stenosis to unveil its function and anatomy, treatment innovations and strategies tailored for bicuspid aortic stenosis, to clinical outcomes. Herein, we introduce the 13 articles collected in this Research Topic (Table 1).

**Table 1:** Information and highlights of the 13 articles in the research topic.

Authors	Key challenges in the field	Objectives of the study	Highlights of the study
Jacob Gutierrez et al.	Congenital heart defects, particularly left-sided anomalies, including BAV, occur in about 30% of individuals with TS and are the leading cause of premature mortality. Despite the significant prevalence of BAV in TS, there has been limited exploration of the potential role of epigenetic regulation in the development of this condition and in TS.	To identify DNAm alterations associated with TS BAV as well as between TS and euploid females with BAV, and detect possible epigenetic modifications in BAV-associated genes and pathways that may further explain the high incidence of BAV and aortopathy in TS	The current study investigates the genomic contributions to the higher susceptibility to BAV in TS, thereby highlighting the probable involvement of epigenetic regulations in the development of both TS and BAV.
Shinjeong Song et al.	Under echocardiographic surveillance, many BAVs are diagnosed without significant valve dysfunction. However, there is limited data is available regarding the progression and outcomes of non-dysfunctional BAV.	To determine the incidence of aortopathy at initial diagnosis and characterize aortic complications among patients with non-dysfunctional BAV vs. dysfunctional BAV, further assess the progression of valvular dysfunction and aortopathy in non-dysfunctional BAV based on a large Korean BAV registry	This study, conducted in Korea, highlights that a significant proportion of individuals with BAV without any significant valvular dysfunction also exhibit aortopathy, which increases the likelihood of AA dilation and subsequent need for aortic operation compared to those without aortopathy. Moreover, the results suggest that most non-dysfunctional BAVs maintain normal valvular function for up to 6 years, providing evidence to support the clinical management of non-dysfunctional BAVs in terms of valvular replacement.
Constance G. Weismann et al.	BAV is the most common congenital cardiac anomaly and has been linked to aortopathy, increased aortic stiffness, and diastolic dysfunction. However, the underlying mechanisms and the impact of age on BAV-associated complications are not yet fully understood.	To characterize arterial and cardiac function, their correlation, and the effect of age in children and adults with a history of BAV by a multimodal approach	This study highlights that children with BAV can experience diastolic dysfunction, which progressively worsens with age, mainly due to reduced ascending aortic distensibility. As a result, these findings shed light on the mechanisms of vascular and ventricular dysfunction in BAV populations, as well as the effect of age.
Mi Chen et al.	According to the current practice guideline, patients with BAV and significant valve dysfunction should undergo ascending aortic replacement if their aortic diameter exceeds 45 mm. However, it is uncertain whether patients with dilated aortas but without significant valvular dysfunction require concomitant AVR.	To compare the perioperative and follow-up benefits and risks of IR vs. PR for BAV-related aortopathy	This study proposes that IR is a better treatment option than PR for patients with BAV-related aortopathy, suggesting a minimum cutoff of 40 mm of aortic diameter for patients with “valve type” and 52 mm for those with “aorta type.” This provides a reference for clinical practice, particularly for patients without significant valvular dysfunction.
Nils Perrin et al.	The impact of BAV morphology on TAVR outcomes remains poorly investigated due to the lack of pivotal randomized trials comparing TAVR with surgery that include BAV. However, data from registries and observational studies that include highly selected patients have shown promising results of TAVR in BAV populations.	To describe anatomical and pathophysiological characteristics of BAV, discuss the main aspects to assess diagnostic imaging modalities, and give an overview of TAVR outcomes and technical considerations specific to BAV morphology in this review	This study provides a review of the anatomical and pathophysiological characteristics of BAV, the main aspects to assess diagnostic imaging modalities, and technical considerations and outcomes specific to BAV morphology with regards to the TAVR procedure.
Giulia Costa et al.	With BAV affecting approximately 1–2% of the population, it is possible that an increasing number of patients with degenerated BAV may eventually require TAVR during the course of their disease. However, BAV presents a challenge due to its unique anatomical features and the absence of consensus on the optimal sizing strategy.	To review the peculiar aspects of BAV and to discuss and compare the currently available sizing methods	This review provides an overview of available sizing methods for the BAV population with regards to the TAVR procedure, as well as ways to optimize procedural outcomes.
Yung-Tsai Lee et al.	According to current guidelines, TAVR should be performed on only selected patients with BAV and AS. However, it is crucial to identify the important factors that affect long-term outcomes in patients with BAV who undergo TAVR.	To identify what the truly important factors are that determine the device success and long-term outcomes in patients with BAV undergoing TAVR	This study provides the first report of the prevalence of BAV referred to TAVR in Taiwan and identifies predictors of prognosis. With the novel sizing method (Wei's Method), safer prosthesis implantation could be achieved when using a balloon-expandable valve.
Jiajun Zhang et al.	Studies on the association of Sievers BAV morphology with conduction disorders after TAVR have not reached consensus.	To pool and analyze about post-TAVR conduction abnormalities and their association with Sievers BAV morphology	This pooled analysis firstly focuses on the association of Sievers BAV morphology with post-TAVR conduction disorders, revealing higher risk of post-TAVR PPI and conduction disorders in type 1 BAV compared with type 0.
Yuchao Guo et al.	NOCDs, including complete left bundle branch block and high-grade atrioventricular block, remain the most common complication after TAVR. However, there is limited data on predictors and strategies to decrease NOCDs in severe AS patients with BAV.	To evaluate the predictors of NOCDs in BAV patients using self-expanding valves and identify modifiable technical factors	This study provides a predictive model for NOCDs after TAVR based on the BAV population receiving self-expandable valves from seven centers in China, providing robust evidence for clinical management to decrease the risk of NOCDs after TAVR.
Gangjie Zhu et al.	SLT is an important sequela that compromises the durability of the bioprosthetic valve of TAVR. Moreover, no studies have compared the SLT detected by CT and its clinical implications and prognoses in patients with BAV and TAV.	To retrospectively assess the SLT defined by the CT in the BAV and TAV stenotic patients	This study presents novel findings indicating a comparable occurrence rate of SLT in BAV patients who received TAVR in a single center, and a similar set of predictors compared to those of TAV patients.
Yi Zhang et al.	The absence of specific guidelines and practical recommendations for TAVR in the BAV population emphasizes the urgent need for a reliable evaluation of the effectiveness and safety of TAVR procedures in these patients.	To conduct a systematic review and meta-analysis of clinical adverse events in patients undergoing TAVR with BAV versus TAV anatomy and the efficacy of BE vs. SE valves stratified into early- and new-generation devices, as well as differences of prosthetic geometry on CT between BAV and TAV and BAV morphological presentations in included studies	This meta-analysis provides an up-to-date synthesis of the most extensive evidence on TAVR in patients with BAV. The findings indicate a higher risk of procedural and 30-day adverse events among BAV patients undergoing TAVR when compared to TAV patients, but a more significant benefit in terms of mortality.
Yu Du et al.	TAVR has achieved satisfactory outcomes in selected patients with BAV, predominately type 1 BAV (∼90%). However, there is limited research on the safety and efficacy of TAVR in type 0 BAV.	To compare procedural and 30-day outcomes after TAVR between type 0 and type 1 BAV through a systematic review and meta-analysis	This study conducted the first meta-analysis comparing the procedural and clinical outcomes of TAVR in patients with Sievers type 0 and type 1 BAV, indicating comparable procedural and 30-day outcomes.
Kyu Kim et al.	The population is aging, and in the last two decades, advances in multimodal imaging and transcatheter valve intervention for BAV have been remarkable.	To investigate temporal trends in demographic characteristics, use of multimodal imaging, treatments, and outcomes in patients with BAV from a large Korean registry	This study aims to provide a systematic description of temporal changes and trends in patient characteristics, valvular function, diagnosis, treatment, and outcomes among patients with BAV from a single tertiary center over the past two decades. These findings will be a valuable reference for further diagnostic and treatment advances.

TS, Turner syndrome; BAV, bicuspid aortic valve; DNAm, DNA methylation; AA, ascending aorta; AVR, aortic valve replacement; IR, integrated aortic-valve and ascending-aortic replacement; PR, partial replacement; TAVR, transcatheter aortic valve replacement; AS, aortic stenosis; PPI, permanent pacemaker implantation; NOCD, new-onset conduction disturbance; SLT, subclinical leaflet thrombosis; CT, computed tomography; TAV, tricuspid aortic valve; BE, balloon expandable; SE, self-expanding.

## Genomic issues in congenital BAV

The genetics behind BAV are acknowledged to be different from a normal tricuspid aortic valve (TAV), as patients with BAV may develop aortic malformations, valvular dysfunctions, or symptoms at a younger age. Several genomic mutations have been found to be associated with BAV, such as mutations in *NOTCH1*, *ROBO4*, etc. (1). In this research topic, Jacob Gutierrez et al. identified that patients with Turner syndrome (TS), a rare cytogenetic disorder presenting a 60-fold increased risk of BAV compared to the general population, have differentially methylated regions (DMRs) encompassing *MYRF* and enrichment for genomic targets, including genes in *NOTCH1* and the downstream gene *MYH11* in those with concomitant BAV. These DMRs in TS appeared to contribute to both BAV development and BAV-associated aortopathy, adding evidence in the genomic etiology of congenital BAV.

## Natural disease course of BAV

With the generalization of echocardiographic surveillance, the diagnosis of non-dysfunctional BAV (BAV without significant aortic stenosis or aortic regurgitation) is increasing. Based on a BAV registry enrolling patients from a single hospital in Seoul, Shinjeong Song et al. found that patients with non-dysfunctional BAV, especially the true BAV, were more likely to be considered as candidates for aortic surgery due to the progression of ascending aortic dilatation. In addition, most non-dysfunctional BAVs could still maintain normal valve function 6 years after their initial diagnosis. In patients with non-dysfunctional BAV, initial BAV function and degree of aorta dilatation might be important factors for disease progression and prognosis.

## Aortopathy in BAV

Aortopathy is common in the BAV population and may predispose to aortic stiffening, dilation and dissection. Despite controversies, aortic stiffening may lead to heart failure through arterio-ventricular interaction (2, 3). In this research topic, Constance G. Weismann et al. used a multimodal method to reveal that ascending aortic distensibility appears to be the most important predictor of diastolic dysfunction in the BAV population, with increased proximal aortic stiffness and wave reflection in both children and adults. Therefore, timely management of proximal arterial stiffness may be a target to prevent further diastolic dysfunction in the BAV population.

Concomitant aortic dilatation is present in about 20%–40% of BAV patients, which may be secondary to abnormalities of the aortic media (4, 5). Currently, guideline recommends ascending aortic replacement in dysfunctional BAV with concomitant dilated aorta if the cutoff of 45 mm is reached (6). Mi Chen et al. proposed a classification to describe the BAV-related dilated aortopathy into valve type and aorta type which represents the most dysfunctional part. Integrated aortic-valve and ascending-aortic replacement (IR) was associated with long-term mortality and reoperation benefits compared to partial replacement, with an IR cutoff of 40 mm in the “valve type” and 52 mm in the “aorta type”. This finding provides a preliminary exploration of the surgical therapy in BAV with different types of dilated aortopathy, providing a reference for clinical management.

## TAVR for BAV

Bicuspid aortic stenosis is one of the most encountered complications in patients with BAV, occurring in >20% of high-risk elderly patients undergoing surgery (7). With the advent of transcatheter aortic valve replacement (TAVR), patients with severe aortic stenosis of any surgical risk have an alternative beneficial therapy. However, bicuspid aortic stenosis has long been regarded as a challenging anatomy. Nils Perrin et al. reviewed the BAV population in the setting of TAVR Apart from the most widely known BAV classification proposed by Sievers, several novel classifications have been updated in aim to achieve better description of the anatomy and prediction of interventional outcomes (8–10). Despite the technical improvements in imaging modalities, difficulties remain in TAVR planning and execution for BAV due to its distinctive anatomy and hemodynamics. The eccentricity of the opening orifice, the asymmetric heavy burden of calcium deposition in BAV would increase the risk of device malposition and mal-expansion, annular rupture, etc. These suboptimal interactions could further lead to new-onset conduction disturbances (NOCDs) and subclinical leaflet thrombosis (SLT), impeding the durability of the bio-prosthesis and patient prognosis.

In order to achieve better results, several sizing strategies have been proposed. Giulia Costa et al. have discussed and compared the currently available sizing methods for TAVR in terms of BAV population, such as “annular” sizing, “supra-annular” sizing, “balloon-technique” BAV sizing, “raphe-based” sizing, Casper algorithm and LIRA method. A specific prosthesis sizing method, i.e., the Wei's method was proposed by Yung-Tsai Lee et al. which achieved safe implantation and efficacious performance of Sapien 3 in the BAV population. The different sizing techniques that have emerged have not yet been tested in large trials, and therefore a better understanding of BAV sizing is needed, especially with regard to different types of devices. Despite prosthesis iteration, new-onset conduction disturbances (NOCDs) are one of the most common complications of TAVR with an increased risk of mortality and rehospitalization (11). Sievers type 1 BAV morphology seems to have a higher risk of permanent pacemaker implantation (PPI) and NOCDs after TAVR than type 0, as reported by Jiajun Zhang et al. To best predict NOCDs in BAV after TAVR who received self-expanding valves, Yuchao Guo et al. have built a model including age, oversizing ratio on left ventricular outflow tract and Δcoronal membranous septum minus implantation depth. Moderate reduction of the oversizing ratio may be a feasible strategy to reduce conduction disturbances while maintaining good peri-procedural outcomes in heavily calcified bicuspid anatomy with short membranous septum length. Regarding the incidence of SLT in patients undergoing TAVR, comparable data were observed between BAV and TAV at 30 days or 1 year after TAVR, as reported by Gangjie Zhu et al. providing more specific evidence of SLT in the BAV population.

Studies are encouraging in the light of similar outcomes to TAVR for the BAV versus TAV population (12, 13). Yi Zhang et al. and Yu Du et al. have done meta-analyses focused on the prognosis of TAVR in BAV patients, both of which demonstrated similar in-hospital and 30-day post-TAVR mortality not only between BAV and TAV, but also between Sievers type 0 BAV and Sievers type 1 BAV, despite a higher risk of other procedural complications such as conversion to surgery, valve-in-valve, paravalvular leak, device failure, acute kidney injury, PPI, and stroke. BAV patients showed a lower 1-year mortality after TAVR than TAV in the report. As the application of TAVR in patients with BAV becomes more frequently on a day-to-day basis in clinical practice, consensus and studies aim for a standardized protocol on TAVR in BAV are being updated (14, 15). Further randomized trials are needed for guidance and standardization of specific peri-operative techniques of TAVR for heterogeneous BAV anatomies, as well as the prognosis in this population.

## Temporal trend of BAV diagnosis and treatment

The demographic characteristics, multimodal imaging, and interventional therapy of BAV have changed over the past two decades. To explore the temporal trends of the aforementioned aspects of the BAV population, Kyu Kim et al. analyzed data from a large Korean registry, and revealed a significant temporal increase in both the age of initial diagnosis and indexed intervention or surgery in the BAV population. Over time, the proportions of non-dysfunctional BAV and significant aortic stenosis increased, while those of significant aortic regurgitation and infective endocarditis decreased. An increase in the use of bioprosthetic valves and TAVR, and survival improvements in BAV were observed.

In summary, the 13 articles in this Research Topic presented the latest advances in the aforementioned aspects of BAV. These discoveries help to better understand and guide clinical practice in this population. However, the conclusions need to be further validated by larger studies and randomized trials in view of the limitations caused by their small size and non-randomized natures.
